# Improved Chemical and Radiochemical Synthesis of Neuropeptide Y Y_2_ Receptor Antagonist *N*-Methyl-JNJ-31020028 and Preclinical Positron Emission Tomography Studies

**DOI:** 10.3390/ph17040474

**Published:** 2024-04-08

**Authors:** Inês C. F. Fonseca, Mariana Lapo Pais, Fábio M. S. Rodrigues, José Sereno, Miguel Castelo-Branco, Cláudia Cavadas, Mariette M. Pereira, Antero J. Abrunhosa

**Affiliations:** 1 CIBIT/ICNAS, Institute for Nuclear Sciences Applied to Health, University of Coimbra, 3000-548 Coimbra, Portugal; inesfonseca@icnas.uc.pt (I.C.F.F.); marianalapopais@gmail.com (M.L.P.); josesereno@uc.pt (J.S.); mcbranco@fmed.uc.pt (M.C.-B.); 2 Faculty of Pharmacy, University of Coimbra, 3000-548 Coimbra, Portugal; ccavadas@ci.uc.pt; 3 ICNAS Pharma, University of Coimbra, 3000-548 Coimbra, Portugal; 4 Coimbra Chemistry Centre, University of Coimbra, 3000-548 Coimbra, Portugal; fabio.rodrigues@uc.pt (F.M.S.R.); mmpereira@qui.uc.pt (M.M.P.); 5 Faculty of Medicine, University of Coimbra, 3000-548 Coimbra, Portugal; 6 CNC—Center for Neuroscience and Cell Biology, University of Coimbra, 3004-504 Coimbra, Portugal; 7 CIBB—Centre for Innovative Biomedicine and Biotechnology, University of Coimbra, 3004-531 Coimbra, Portugal

**Keywords:** NPY Y2 receptor, radiotracer synthesis, continuous flow synthesis, PET imaging, *N*-methyl-JNJ-31020028

## Abstract

Neuropeptide Y (NPY) is one of the most abundant peptides in the central nervous system of mammals and is involved in several physiological processes through NPY Y_1_, Y_2_, Y_4_ and Y_5_ receptors. Of those, the Y_2_ receptor has particular relevance for its autoreceptor role in inhibiting the release of NPY and other neurotransmitters and for its involvement in relevant mechanisms such as feeding behaviour, cognitive processes, emotion regulation, circadian rhythms and disorders such as epilepsy and cancer. PET imaging of the Y_2_ receptor can provide a valuable platform to understand this receptor’s functional role and evaluate its potential as a therapeutic target. In this work, we set out to refine the chemical and radiochemical synthesis of the Y_2_ receptor antagonist *N*-[^11^C]Me-JNJ31020028 for in vivo PET imaging studies. The non-radioactive reference compound, *N*-Me-JNJ-31020028, was synthesised through batch synthesis and continuous flow methodology, with 43% and 92% yields, respectively. *N*-[^11^C]Me-JNJ-31020028 was obtained with a radiochemical purity > 99%, RCY of 31% and molar activity of 156 GBq/μmol. PET imaging clearly showed the tracer’s biodistribution in several areas of the mouse brain and gut where Y_2_ receptors are known to be expressed.

## 1. Introduction

Neuropeptide Y (NPY) is a conserved 36-amino acid peptide belonging to a family of regulatory peptides, including peptide YY (PYY) and pancreatic peptide (PP). It stands as one of the most prevalent peptides within the central nervous system of mammals [1,2]. NPY is involved in processes of energy homeostasis, water consumption, circadian rhythms, sleep, learning, memory, emotional regulation and angiogenesis [3]. NPY’s physiological processes are mediated through the activation of different subtypes of NPY receptors (Y_1_, Y_2_, Y_4_ and Y_5_) that are expressed in both central and peripheral nervous systems and belong to the G-protein coupled receptors (GPCR) superfamily [4]. Notably, the Y_2_ receptor has gained some interest over the years. It has been linked to the physiological processes of feeding behaviour, anxiety, neuronal excitability, angiogenesis, circadian rhythm, alcohol dependence, cognitive processes and locomotor activity [5,6,7,8,9]. Additionally, the Y_2_ receptor is mainly pre-synaptic and has an autoreceptor role, as it inhibits the release of NPY and other neurotransmitters [10]. Despite the extensive study and characterisation of the Y_2_ receptor, its neuromolecular implication in several diseases is not yet fully understood. Developing a suitable PET imaging biomarker could provide a platform to study the underlying mechanisms of these and other diseases and identify possible drug targets. Winterdahl et al. reported the development of an *N*-[^11^C]Me-JNJ-31020028 PET tracer for the Y_2_ receptor and brain imaging in living pigs [11]. The favourable attributes of the Y_2_ receptor antagonist JNJ-31020028 were leveraged, such as its high affinity and selectivity towards the Y_2_ receptors [12], and the radiolabelling was performed using [^11^C]CH_3_I. The radiotracer permeated the blood–brain barrier, and its binding was in agreement with the anatomical distribution of the Y_2_ receptors described in reference [11]. It also showed rapid distribution throughout the brain and slow metabolization in the bloodstream. However, these studies were mainly focused on the binding affinity of *N*-[^11^C]Me-JNJ-31020028 and its interaction with the Y_2_ receptor and were not continued. Despite the importance of these studies, we aimed to deepen the knowledge of the Y_2_ receptor’s biodistribution as it can provide insights into its relevance in health and disease. 

The development of PET tracers involves the optimisation of several key steps, such as the radiolabelling reaction, purification and reformulation conditions and the quality control of the final product. A non-radioactive reference compound is needed for the labelled product’s radiochemical identification and HPLC control analysis. Moreover, this standard compound is required to calculate the labelled product’s molar activity, which is relevant for potential receptor occupancy and saturation and is defined as the measured radioactivity per mole of compound [13]. When this standard compound is not available commercially, its synthesis is required. Thus, one can obtain the desired non-radioactive reference compound by conventional batch synthesis or use a continuous flow methodology to produce the compound on a larger scale. Continuous flow or microfluidic technology is based on conducting chemical reactions in a continuous stream within narrow channels that allow unique control over the reaction conditions. This technology allows the use of lower reaction volumes, higher pressures and better temperature control, leading to reduced reaction times and better product selectivity/yield with the benefit of being easy to automate [14]. Not only is this technology highly used in organic synthesis but also in PET radiochemical preparations. The characteristics of this methodology present an improvement in PET chemistry in several key steps that often are problematic when conducting radiosynthesis, such as the need for quick reactions due to short-lived radionuclides, namely ^18^F (110 min half-life) and ^11^C (20 min half-life), and reduced consumption of necessary and expensive reagents [15,16,17]. Consequently, continuous flow technology has been implemented to synthesise different radiopharmaceuticals. For instance, in carbon-11 radiochemistry, microfluidics has been used in ^11^C-methylations and ^11^C-carbonylations reactions with some promising results [17,18] and radiopharmaceuticals like ^11^C-DASB [19], ^11^C-raclopride [20] and ^11^C-flumazenil [21] have been prepared through this technology, highlighting the potential of the use of microfluidics in PET radiotracer synthesis. 

Hereby, we present the chemical and radiochemical developments of the Y_2_ receptor antagonist *N*-Me-JNJ31020028. First, *N*-Me-JNJ31020028 was synthesised, isolated, characterised and used as the analytic standard for the following studies. Then, the development of a continuous flow synthetic methodology for the preparation of the non-radioactive compound was performed, which enabled synthesising the reference compound on a larger scale and represented a valuable preliminary study to implement this type of technology in radiotracer production. Subsequently, we described the development and implementation of a new process of radiosynthesis of the PET tracer *N*-[^11^C]Me-JNJ31020028 and the first PET images and biodistribution studies, to our knowledge, in healthy C57BL/6N mice representing a solid baseline of expertise for future research within mouse models.

## 2. Results and Discussion

### 2.1. Batch Synthesis

**Scheme 1:**
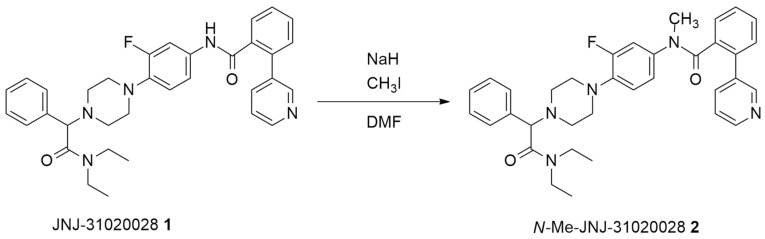
Batch synthesis of *N*-Me-JNJ31020028.

**Figure 1:**
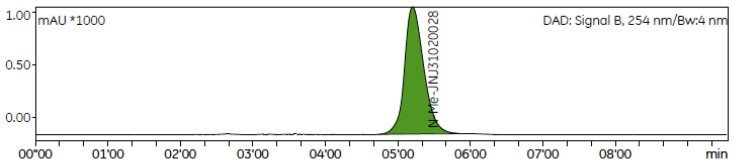
Analytical HPLC of *N*-methylated reference compound **2**. Rt: 5.12 min. (See Supplementary Materials for further analytical information, Figure S8).

Aiming to prepare pure *N*-Me-JNJ31020028 **2** as an analytic standard, the studies began with the batch *N*-methylation of precursor JNJ-31020028 **1** (Scheme 1). The synthesis was performed via JNJ-31020028 **1** reaction with 1.5 equiv. of iodomethane and 1.5 equiv. of sodium hydride (60% mineral oil dispersion) in DMF at room temperature for 1 h and 45 min. The needed product was isolated by flash chromatography using a gradient of dichloromethane and MeOH/NEt_3_ mixture as eluent. *N*-Me-JNJ-31020028 **2** was obtained with a 42% isolated yield. The purity of the *N*-methylated compound **2** was analysed through HPLC (Figure 1), where it was observed to have chemical purity > 99%. It was also fully characterised by 1D/2D NMR spectroscopies and HRMS; the data are available in the Supplementary Materials (Figures S1–S7).

### 2.2. Continuous Flow Synthesis

**Figure 2:**
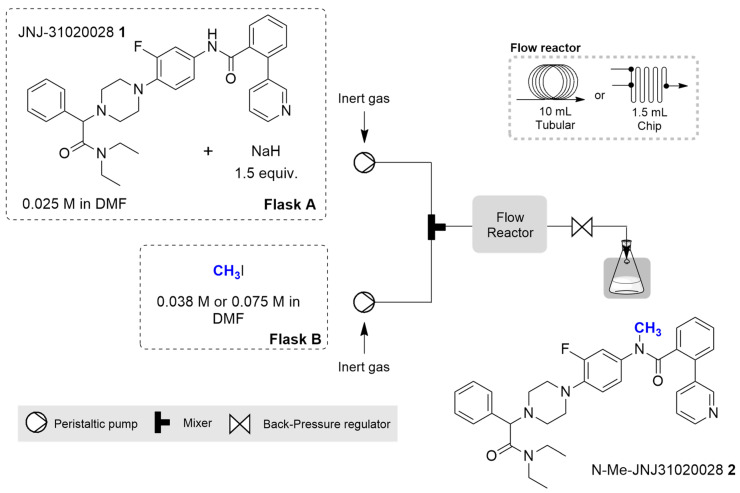
Representation of the continuous flow synthesis apparatus configuration for the synthesis of *N*-Me-JNJ-31020028. Flask A contains a solution of precursor **1** (1 equiv., 0.025 M) and NaH (1.5 equiv.) in DMF; flask B contains a solution of CH_3_I (1 equiv., 0.038 M or 3 equiv., 0.075 M).

Continuous flow methylation of precursor **1** was performed using the same synthetic strategy used for the batch preparation but taking two approaches: one using a tubular reactor, ideal for scaling-up of reactions, and the other using a chip reactor, more suitable for transposition of the process to the radiolabelling facility. The general apparatus configuration consisted of an E-Series Vapourtec’s flow chemistry apparatus equipped with an inert gas kit, V3 pumps, a continuous flow reactor (either a 10 mL tubular or a 1.5 mL chip reactor) and a collection valve. Two peristaltic pumps were used to pump a 0.025 M solution of (**1**) with 1.5 equiv. of sodium hydride (flask A) and an iodomethane 0.038 or 0.075 M solution (flask B), as depicted in Figure 2. 

Each experiment was performed by pumping the solution in flasks A and B at a constant flow rate through the flow chemistry reactor (thermostated at 25 or 40 °C). When the steady state was reached, the reaction crude flow stream was collected, and the conversion and yield of **2** were determined by HPLC analysis. The reaction parameters regarding the equivalents of CH_3_I, temperature and residence time were optimised and the results are presented in Table 1 and Table 2.

The studies started by using a tubular flow reactor (10 mL) (Table 1). Firstly, we tested different residence times of CH_3_I (1.5 equiv., 0.038 M), such as 25, 10, 5 and 3 min (Table 1, Entries 1–4), at 25 °C. The best result was achieved when the residence time was set to 5 min with complete conversion of precursor **1** and 90% yield of compound **2** (Table 1, Entry 3). Increasing the residence time to 10 min did not improve the yield (Table 1, Entry 2), and at 25 min, the conversion and yield decreased to 82% and 64%, respectively (Table 1, Entry 1). This result may be attributed to the degradation of **2** at higher residence times. Complete conversion was obtained with 3 min of residence time, but a decrease in yield to 88% was observed (Table 1, Entry 4). Then, with 5 min of residence time, the temperature was increased to 40 °C, and a slight yield improvement (92%) was obtained (Table 1, Entry 5). Next, we evaluated the increase in CH_3_I from 1.5 (0.038 M) to 3 equiv. (0.075 M) at either 25 or 40 °C. In this case, an increase in side product formation (representative HPLC analysis in the Supplementary Materials, Figure S9) was observed through the decree of product yield of 81 and 77%, respectively (Table 1, Entries 6–7). To summarise, 5 min of residence time, 25 °C and 1.5 equiv. of CH_3_I (Table 1, Entry 3) were shown to be the best reaction conditions to perform the methylation reaction in which it was possible to obtain *N*-Me-JNJ-31020028 **2** with a productivity of 1.4 mmol/h. 

Aiming to transpose the continuous flow synthesis conditions of **2** to the radiosynthesis protocols that use small-scale reactors and loops, we also evaluated the use of a chip flow reactor using the standard apparatus configuration equipped with a 1.5 mL chip reactor (Table 2). Using the previous best reaction conditions (1.5 equiv. of CH_3_I, at 25 °C and 5 min of residence time), compound **2** was obtained with 66% conversion and 60% yield (Table 2, Entry 1). A significant improvement was achieved by increasing the temperature to 40 °C, and **2** was obtained with 94% conversion and 88% yield (Table 2, Entry 2). The best results were achieved when using 3.0 equiv. of CH_3_I at 40 °C, as the *N*-methylated product **2** was obtained with 95% conversion and 89% yield (Table 2, Entry 3). Furthermore, it should be noted that in PET chemistry, the methylating agent is in the gas phase instead of iodomethane in solution, which was used in these studies. For that reason, we attempted to simulate the reaction of precursor **1** with gaseous CH_3_I. Liquid CH_3_I was introduced in a 55 °C bath, which led to its vaporisation. The gas was then pumped to the flow reactor at a 1:1 volumetric ratio related to precursor **1**, and the reaction was performed at 40 °C with 5 min of residence time, obtaining 98% conversion and 67% yield (Table 2, Entry 4). This result reveals the potential to implement flow chemistry systems in the radiolabelling protocols for the production of ^11^C-labeled PET radiotracers, in particular, *N*-[^11^C]Me-JNJ-31020028.

### 2.3. Radiochemistry

To implement a protocol for the radiosynthesis of *N*-[^11^C]Me-JNJ-31020028, several reaction conditions, such as the base, reaction temperature and time and the use of stirring were tested and optimised for the radiochemical methylation of **1** (Table 3). The methylating agent [^11^C]CH_3_I was obtained through the cyclotron-produced [^11^C]CO_2_ transformation through the ‘gas-phase’ method [22,23]. The conditions presented in entries 1–8 in Table 3 were created in a small reaction vessel, in which the methylating agent was trapped in a solution containing precursor **1** in the respective solvent and base. Initially, we tried to reproduce the conditions of JNJ-31020028 radiolabelling with [^11^C]CH_3_I, already reported in the literature [11], by using sodium hydride (NaH, 60% dispersion in mineral oil) as a base, a mixture of DMF-THF as the solvent and with the time of reaction set to 3 min (Table 3, Entry 1). According to HPLC analysis, this led to a very high product percentage with an RCY of 42%. Some further optimisations using this base were also made but did not lead to significantly better results regarding **[^11^C]2** formation (Table 3, Entries 2–4). 

Nonetheless, even though the sodium hydride base is a very suitable base for the deprotection of the amide group of the JNJ-31020028 (**1**) precursor, its high reactivity represents a threat to the good handling of the equipment used in radiosynthesis. Its corrosiveness can reduce the lifetime of the synthesis apparatus and increase radiosynthesis failure rates. After experiencing some troubles due to this reason, we decided to use an alternative approach. Hence, the optimised alternative radiosynthesis protocol (Table 3, Entries 5–9) was based on ^11^C-methylation protocols developed in-house. We used an aqueous solution of NaOH 5M as the base and tested the differences between temperature and stirring effects. The reaction time was set for 5 min conventionally as NaOH reactivity is not as high as NaH, adding to the fact that radiolabelling with very short half-lived radioisotope ^11^C requires rapid reaction times. In these optimisations, we observed that the no stirring effect led to higher product percentages (Table 3, Entries 5 and 7) and slightly better radiochemical yields than when stirring was used (Table 3, Entries 6 and 8). This led to testing this radiolabelling reaction using the “in loop” method. An HPLC loop that is connected to an HPLC purification system is coated with the solution of precursor and base in a solvent before [^11^C]CH_3_I trapping. After the reaction, the crude is directly purified through semi-preparative HPLC (Table 3, Entry 9). As is possible to see, the ‘in loop’ method generated **[^11^C]2** in a high product percentage, with an RCY of 31%. 

**Figure 3:**
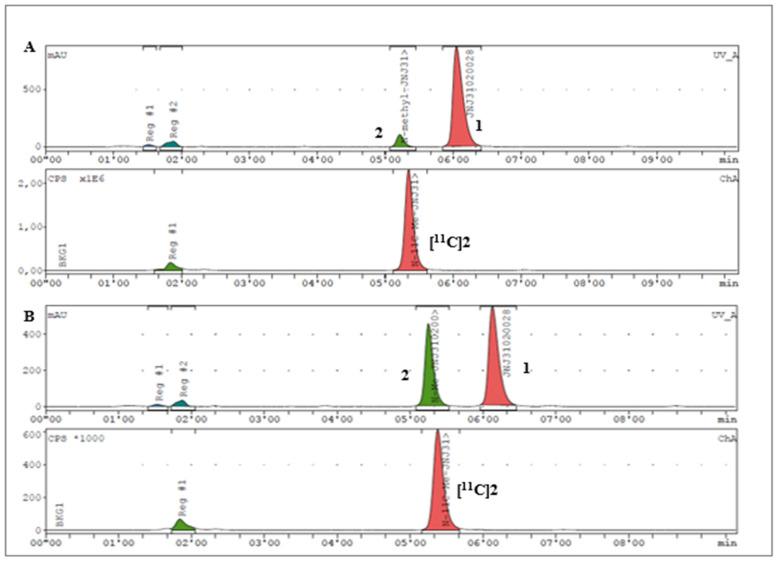
Analytical HPLC chromatograms: (**A**) radiolabelling reaction crude analysis: **1** Rt = 6.03 min; **2** Rt = 5.13 min; **[^11^C]2** Rt = 5.21 min. (**B**) Co-injection of the same mixture reaction used in (**A**) and standard reference compound **2**.

Regarding the radiochemical identification of the compound **[^11^C]2**, the previously synthesised standard compound **2** was used to confirm that the ^11^C-labeled product was the desired *N*-[^11^C]Me-JNJ31020028 compound. As shown in Figure 3, the achievement of the tracer *N*-[^11^C]Me-JNJ31020028, **[^11^C]2**, was confirmed through the co-injection of the standard compound **2** with the samples of the crude mixture obtained in the radiolabelling studies.

Within these results, we selected the ‘in loop’ method to produce the radiotracer. One crucial reason for choosing this method was the logistics and necessary module settings to perform these reactions. The ‘in loop’ method allows for the radiolabelling reaction and consequent purification through a semi-preparative HPLC system in a more effective manner, as both systems are connected. Performing the radiolabelling reaction in a vessel means that the crude mixture would need to be transferred to a different hot cell so that purification through the HPLC system can be performed. Thus, this methodology presents some limitations since it would lead to several activity losses during the whole process. Therefore, using the ‘in loop’ method, this production starts with the transformation of [^11^C]CO_2_, produced in a cyclotron, in [^11^C]CH_3_I, through the Synthra [^11^C]Choline commercial module, followed by the transfer of activity to the loop, which is already coated with the solution of precursor **1** in DMF with NaOH 5M (aq. sol.). The reaction ‘in loop’ continues for 5 min. Afterwards, the mixture is transferred to the HPLC equipped with a semipreparative column, where the desired product is purified. After collection, reformulation of the product to an adequate injectable solution is performed. 

This method allowed for the successful and reproducible synthesis of radiotracer *N*-Me-JNJ31020028 **[^11^C]2** with radiochemical purity > 99% (*n* = 3), a radiochemical yield of 31% (*n* = 3) and molar activity of 156.62 ± 44.62 GBq/μmol (*n* = 3). 

After each production, quality control tests of the final product were performed according to the procedures described in the European Pharmacopoeia (Eur. Ph.) [24]. Table 4 presents the specifications and results of the quality control for three validation batches of *N*-[^11^C]-Me-JNJ31020028 injectable solution. The chemical and radiochemical purity specifications were stated internally at ICNAS Pharma, according to the International Council for Harmonisation of Technical Requirements for Pharmaceuticals for Human Use (ICH) guidelines [25].

The radiosynthesis of *N*-[^11^C]-Me-JNJ31020028 using the continuous methodology is dependent on the implementation of the continuous flow processes technology into the radiochemical laboratory, which was not completed in the timeframe of this study.

### 2.4. PET Imaging

**Figure 4:**
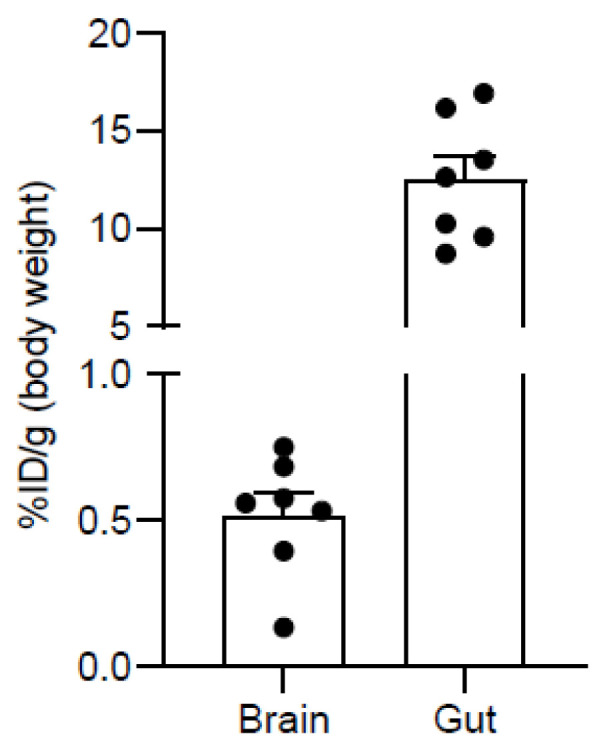
Uptake of *N*-[^11^C]Me-JNJ-31020028 in the gut and brain in mice 30–50 min post-injection. Values are expressed as percentage injected dose per gram (%ID/g) (mean ± SEM, *n* = 7, see Supplementary Materials).

PET/MRI images of *N*-[^11^C]Me-JNJ-31020028 and biodistribution studies were performed in healthy C57BL/6N mice. The radiotracer **[^11^C]2** injectable solutions were administered intravenously to the mice, previously anaesthetised, and the mice were put in the MicroPET scanner for PET imaging acquisition. The MRI acquisition was performed before injection due to the necessity of using equipment different from MicroPET. The in vivo biodistribution studies were performed right after injection and immediately showed a higher accumulation of radiotracer in the gut (12.57 ± 1.27%ID/g) in comparison with the radiotracer’s accumulation in the brain (0.5187 ± 0.08%ID/g) (Figure 4). 

**Figure 5:**
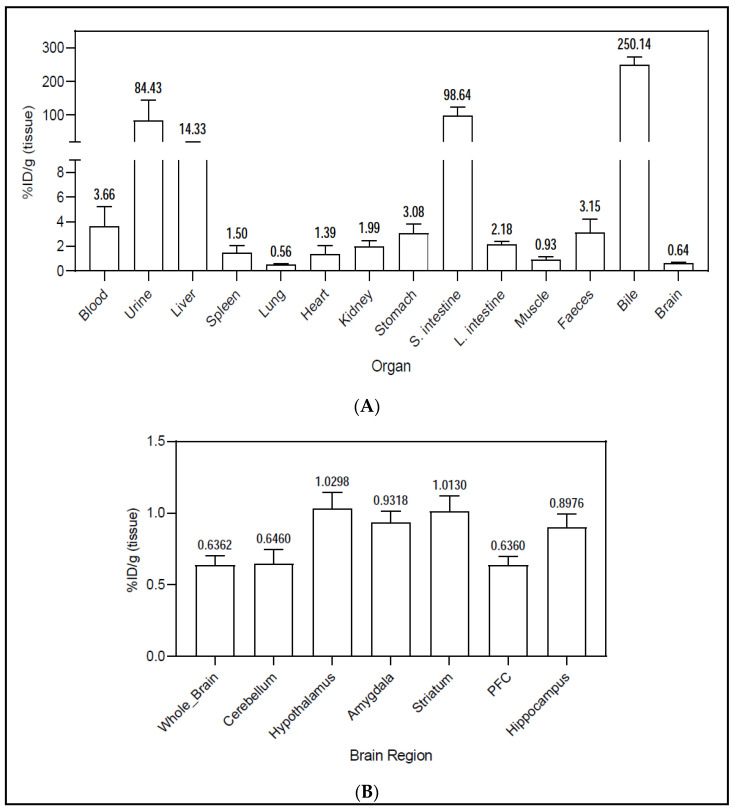
Ex vivo biodistribution (gamma counting) of *N*-[^11^C]Me-JNJ-31020028 in (**A**) the whole body and (**B**) the brain. Values are expressed as percentage injected dose per gram (%ID/g) of tissue (mean ± SEM, *n* = 5, see Supplementary Materials).

The ex vivo biodistribution studies were performed one hour after the intravenous injection of the **[^11^C]2** injectable solution. For these studies, transcardiac perfusion with saline was performed, eliminating blood traces from the organs. These studies showed an uptake in bile (250.1 ± 23.97%ID/g), showing that the radiotracer is mainly eliminated through the hepatobiliary system (Figure 5A). Urinary excretion also occurred, though less radioactivity was found in the urine (84.43 ± 59.41%ID/g). The small intestine (98.64 ± 25.57%ID/g) and liver (14.33 ± 6.39%ID/g) were the organs with major uptake of the radiotracer. The high uptake in the small intestine is explained by the high density of Y_2_ receptors that exist in this organ [27,28,29], while the uptake in the liver is related to the metabolization of the tracer. Moderately low uptake was found in the stomach (3.082 ± 0.7135%ID/g), large intestine (2.18 ± 0.21%ID/g) and kidneys (1.99 ± 0.49%ID/g). Our studies also showed moderate levels of the radiotracer in the blood (3.66 ± 1.55%ID/g), suggesting a slow metabolization of the radiotracer in the bloodstream, which was also mentioned by Winterdahl et al. [11].

In the brain, the areas with most radiotracer uptake were the hypothalamus (1.03 ± 0.11%ID/g), striatum (1.013 ± 0.11%ID/g), amygdala (0.9318 ± 0.08%ID/g) and the hippocampus (0.8976 ± 0.09%ID/g) (Figure 5B), which is in accordance with the Y_2_ receptor density in the brain previously described in reference [30]. The cerebellum (0.646 ± 0.10%ID/g) and pre-frontal cortex (PFC) (0.636 ± 0.06%ID/g) were the brain regions with the least radiotracer uptake.

**Figure 6:**
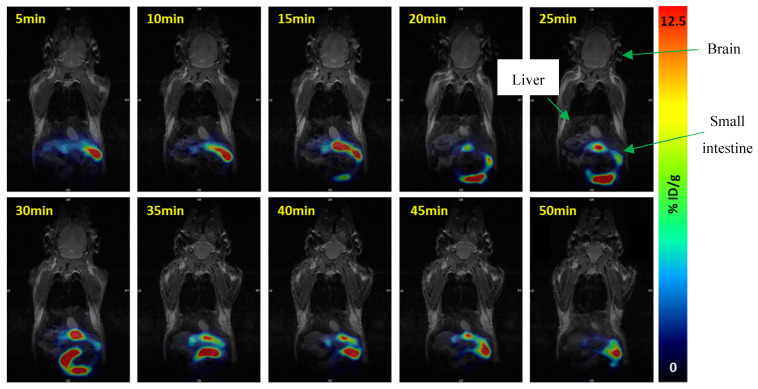
Whole-body distribution of *N*-[^11^C]Me-JNJ-31020028 in healthy C57BL/6N mice over time (PMOD, v3.6; PMOD. FUSION tool from PMOD was used to co-register PET images with the anatomical MRI).

The PET images were acquired and fused with the MRI image to better elucidate the areas in which the radiotracer was bound to the Y_2_ receptor. Figure 6 presents the whole-body distribution PET image of *N*-[^11^C]Me-JNJ-31020028 over time. Even though the radiotracer has whole-body distribution, it is only possible to see the radiotracer’s accumulation in the gut, reaching its peak in between 25 and 30 min. This may not mean that the radiotracer only accumulates in the small intestine. However, it accumulates in a higher density than in other organs and the brain, confirmed by the ex vivo biodistribution depicted in Figure 5. This higher accumulation of the tracer in the small intestine is observed in both PET images and ex vivo results, in which, in the latter, the sample analysed was only the intestine wall. Therefore, these results represent the uptake of the tracer by the high density of Y_2_ receptors in the small intestine and are not due to excretion.

**Figure 7:**
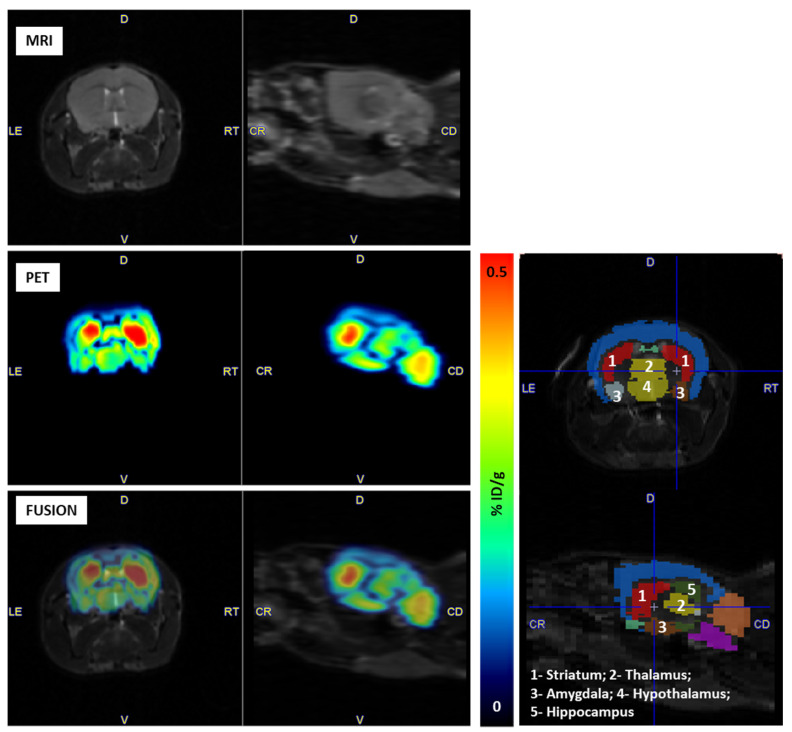
Brain distribution of *N*-[^11^C]Me-JNJ-31020028 in healthy C57BL/6N mice. Left: MRI and PET images and fusion of both. Right: Identification of brain areas with PMOD (PMOD, v3.6; PMOD. FUSION and PNOR tools from PMOD were used to co-register PET images with the anatomical MRI and to define volumes-of-interest (VOIs) based on an anatomical ATLAS, Mouse (Ma-Benveniste-Mirrione) VOI atlas).

Concerning the radiotracer’s accumulation in the brain, it was possible to identify several brain regions with high radiotracer accumulation, such as the striatum, thalamus, amygdala, hypothalamus and hippocampus (Figure 7).

Future work will include the study of the *N*-[^11^C]Me-JNJ-31020028 tracer in pre-clinical models of brain disorders.

## 3. Materials and Methods

### 3.1. Materials 

JNJ-31020028 **1** was commercially obtained from InvivoChem LLC (Libertyville, IL, USA). *N*,*N*-Dimethylformamide, 99.8%, Extra Dry was purchased from Fisher Scientific (Porto Salvo, Portugal). Sodium hydride, 57–63% oil dispersion, was purchased from Enzymatic (Santo Antão do Tojal, Portugal). Sodium hydroxide and iodomethane were purchased from Merck (Lisboa, Portugal). Air- and moisture-sensitive reagents or solutions were handled under nitrogen or argon atmosphere in a vacuum system using Schlenk techniques [31]. All the glassware was dried by heating. The solvents, such as dichloromethane and ethyl acetate, were purified by simple distillation.

### 3.2. Instrumentation

Nuclear magnetic resonance (NMR) spectra were recorded with CDCl_3_ using the Brucker Avance III spectrometer (Department of Chemistry, University of Coimbra), operating at 400 MHz (^1^H-NMR) and 100 MHz (^13^C-NMR). The molecular weights were measured by a high-resolution mass spectrometer belonging to the Unidade de Masas e Proteómica from the University of Santiago de Compostela (Santiago de Compostela, Spain).

### 3.3. Synthesis of N-Me-JNJ-31020028

Batch synthesis: A solution of JNJ-31020028 (1 equiv., 0.18 mmol) and NaH (60% mineral oil dispersion) (1.5 equiv., 10.5 mg) in dried DMF (5 mL) under an inert atmosphere was stirred at ambient temperature for 30 min. Then, CH_3_I (1.5 equiv., 0.26 mmol, 16.4 µL) was added to the reaction mixture. The reaction mixture was stirred for 1 h and 45 min at room temperature and was controlled by analytical HPLC (Agilent 1260 Infinity II LC Series, AgilentSanta Clara, CA, USA), using Phenomenex LUNA 3 µm CN 100 Å, 150 × 3 mm column and eluent 0.1 M ammonium formate aqueous solution pH = 5/Acetonitrile (55:45), at a flow rate of 0.5 mL/min and detection at λ = 254 nm. The crude mixture was dissolved in ethyl acetate and washed with water (3x). The organic phase was then collected and dried over NaSO_4_, filtered and concentrated. The resulting mixture was purified by flash chromatography (PuriFlash XS 420, Interchim, Montluçon, France) using a 15 μm Si-HP puriFlash^®^ column and a gradient of dichloromethane and MeOH/NEt_3_ (7 mL/min). Yield: 42% (43 mg, 0.07 mmol). 

^1^H-NMR (400 MHz, CDCl_3_) *δ*/ppm: 8.51 (d, *J* = 4.0 Hz, 1H), 8.15 (d, *J* = 1.4 Hz, 1H), 7.50 (d, *J* = 7.2 Hz, 1H), 7.41 (d, *J* = 7.8 Hz, 1H), 7.36–7.34 (m, 2H), 7.31–7.23 (m, 5H), 7.21–7.18 (m, 1H), 7.02 (d, *J* = 7.2 Hz, 1H), 6.38 (t, *J* = 9.0 Hz, 1H), 5.84 (dd, *J* = 8.5, 2.4 Hz, 1H), 5.80 (dd, *J* = 13.3, 2.4 Hz, 1H), 4.15 (s, 1H), 3.39–3.30 (m, 2H), 3.13 (s, 3H), 3.23–3.08 (m, 2H), 2.92 (brs, 4H), 2.61–2.52 (m, 4H), 1.00 (t, *J* = 7.1 Hz, 3H), 0.96 (t, *J* = 7.1 Hz, 3H). ^13^C-NMR (100 MHz, CDCl_3_) *δ*/ppm: 170.6, 169.8, 154.3 (d, *J* = 248.1 Hz), 149.5, 148.2, 138.5 (d, *J* = 8.5 Hz), 136.7 (d, *J* = 9.5 Hz), 136.2, 135.8, 135.8, 135.0, 129.9, 129.5, 129.3, 129.2, 128.7, 128.7, 128.3, 123.0, 122.0 (d, *J* = 2.9 Hz), 117.9 (d, *J* = 3.4 Hz), 113.9 (d, *J* = 28.2 Hz), 70.9, 51.2, 50.5, 41.6, 40.8, 37.1, 12.9, 12.4. HRMS (ESI): *m*/*z* calcd. for C_35_H_39_FN_5_O_2_^+^ [M+H]^+^: 580.3082, found: 580.3082.

*Continuous flow synthesis:* Continuous-flow reactions were performed on a Vapourtec E-series (Vapourtec Ltd., Bury St Edmunds, Suffolk, UK) apparatus equipped with chemical-resistant PFA tubing (1 mm ID). Its standard configuration consists of two V3 peristaltic pumps, a tubular (10 mL, 1 mm ID) or microchip reactor (1.5 mL) and a manually adjustable back-pressure regulator (BPR) from Vapourtec (up to 10 bar). An inert gas kit from Vapourtec was used to introduce reagents/solvents under a nitrogen flow. Before all reactions, all channels, pumps and modules were purged with DMF and kept under an inert atmosphere of N_2_. Two solutions were prepared: solution A containing JNJ-31020028 (1 equiv., 0.025 M) and the base (1.5 equiv., 0.037 M) in DMF and solution B containing the methylating agent (CH_3_I) in DMF (1.5 equiv., 0.038 to 3 equiv., 0.075 M). Both solutions were pumped in a 1:1 volumetric ratio into a thermostated reactor using different flow rates. At the end, the reaction crude was collected at the steady state and analysed through the previously described HPLC method. The percentage of the product obtained in each reaction was found through an HPLC calibration curve of *N*-Me-JNJ-31020028.

### 3.4. Radiochemistry

[^11^C]CO_2_ was produced by ^14^N(p,α)^11^C nuclear reaction using a nitrogen gas target pressurised to 18 bar and bombarded with 18 MeV protons produced by the IBA Cyclone^®^ 18/9 cyclotron (IBA, Louvain-La-Neuve, Belgium) at ICNAS Pharma Unipessoal, Lda. Irradiation was performed for 30 min with a beam current of 20 µA. [^11^C]CO_2_ was then converted to [^11^C]CH_3_I through the gas-phase approach, using the Synthra [^11^C]Choline commercial module (Synthra GmbH, Hamburg, Germany) controlled by the software SynthraView version 2.0, by first being reduced to [^11^C]CH^4^ through a nickel catalyst/column, followed by reaction with I_2_ at 750 °C and further recirculation that allowed [^11^C]CH_3_I to be trapped on the Porapak cartridge. Afterwards, [^11^C]CH_3_I was released through a 5 mL/min helium flow to the external reactor or to the loop (Autoloop system, Bioscan Inc., Washington, DC, USA), where the radiolabelling reaction occurred.

[^11^C]CH_3_I was trapped in a solution of JNJ-31020028 (0.5 mg) and NaH (2 mg) or NaOH 5M aqueous solution (3 µL) in DMF (100 µL) for 5 min. The crude mixture was purified by semi-preparative HPLC with an HPLC K-501 pump (Knauer, Berlin, Germany) connected with a UV K-200 detector set at 254 nm (Knauer, Germany), a radioactivity detector and column: Phenomenex LUNA 5 µm CN 100 Å, 250 × 10 mm column; mobile phase: 0.1 M ammonium formate aqueous solution pH = 5/Acetonitrile (55:45), 8 mL/min. The fraction containing *N*-[^11^C]Me-JNJ31020028 was collected between 5 and 6 min into a vial with 10 mL of water and then reformulated. Reformulation proceeded through the IBA Synthera^®^ Extension module (IBA, Belgium) with a disposable kit (Fluidomica, Cantanhede, Portugal) inserted on a reusable cassette support. The aqueous solution was passed through a pre-conditioned Sep-Pak^®^ plus light tC18 cartridge (Waters, Wexford, Ireland). The radiolabelled compound was then released with 1 mL EtOH and 9 mL of saline solution and filtered through a 0.2 µm sterile membrane filter (Minisart^®^, Sartorius Stedim Biotech GmbH, Göttingen, Germany). Quality control of the carbon-11-labelled product solution was conducted through the analytical HPLC (Agilent) with the previously described analytical HPLC method. The ^11^C-labelled product had a retention time of approximately 5 min and was achieved with radiochemical purities of 99%. The carbon-11 radioisotope identity was confirmed by measuring its half-life in the ISOMED 2010 dose calibrator (Nuklear-Medizintechnik GmbH, Dresden, Germany). The residual solvent concentration in the final ^11^C-labelled product solution was monitored through a gas chromatography (GC) system (GC Agilent 6850, Elysia-Raytest Gmbh, Straubenhardt, Germany). The pH was measured using a Quantofix^®^ Relax pH meter (Macherey-Nagel GmbH & Co. KG, Düren, Germany). Bacterial endotoxins were determined with an endosafe^®^-PTS (Portable Test System) spectrophotometer from Charles River Laboratories (Wilmington, MA, USA). Sterility tests were performed at Laboratorios Micro-Bios (Sant Joan Despi, Spain).

### 3.5. Pre-Clinical Studies

*Animals.* Adult male and female C57BL/6N mice aged 7-11 weeks were used. Mice were housed at the Institute of Nuclear Science Applied to Health (ICNAS) animal facility at 22 °C under a 12 h light/dark cycle and provided ad libitum access to food and water. Experimental procedures were reviewed and approved by the Animal Welfare and Ethics Body of ICNAS (ORBEA), approval number: Parecer 1/2018, following the guidelines of the European Community for the use of animals in the laboratory (86/609/EE) and the Portuguese law for the care and use of experimental animals (DL n° 129/92).

*PET/MRI Imaging.* PET/MRI acquisitions were performed at the Institute for Nuclear Sciences Applied to Health (ICNAS) animal facility. During both acquisitions, the animals (*n* = 7) were anaesthetised using 1.5–2% isoflurane, and vital signs, such as temperature and respiratory cycle, were monitored. The radiotracer was administered intravenously (i.v.) in a total injected activity of 12.0 ± 2.9 µCi/g. Mice were placed in a MicroPET scanner based on resistive plate chamber detectors (RPC-PET) [32,33]. Approximately 5 min after tracer injection, a 50 min list mode data acquisition in 3D mode was initiated. After PET acquisition, an anatomical magnetic resonance image (MRI) was performed for co-registration. MRI acquisitions were acquired using a Bruker system (Bruker, BioSpin, Ettlingen, Germany) operated with ParaVision (v6.0.1). Anatomical reference images were obtained by coronal and axial T2-weighted scans. Quantitative imaging processing was performed using PMOD (PMOD, v 3.6; PMOD Technologies, Zürich, Switzerland, RRID: SCR_016547). FUSION and PNOR tools were used to delimitate volumes of interest (VOIs) on the PET images co-registered with the anatomical MRI. Standardised uptake values (*SUVs*) were calculated as [[total positron (β+ radioactivity) concentration in the VOI (Bq/mL)]/[total positron (β+ radioactivity) injected (Bq)/weight (g)]] (Equation (1)) from 30 to 50 min post-injection. The percentage injected dose per gram (%ID/g) was calculated through Equation (2).

(1) $$SUV=\frac{Activity\;concentration\;(MBq/mL)}{Injected\;Dose\;(MBq)}\times Body\;Weight\;(g)$$

(2) $$\%ID/g=\frac{Activity\;concentration\;(MBq/mL)}{Injected\;Dose\;(MBq)}\times 100$$

*Biodistribution.* In another cohort of animals (*n* = 5), ex vivo biodistribution (%ID/g of tissue) was performed by gamma counting through CRC^®^-55t well counter and CRC^®^-55tW radioisotope dose calibrator (Capintec, Ramsey, NJ, USA). Six regions of the brain were also isolated to characterise brain accumulation; the isolation of the specific brain regions was conducted following established protocols [34,35,36,37].

## 4. Conclusions

In this work, we reported an improved radiosynthesis of the PET tracer Y_2_ antagonist *N*-[^11^C]Me-JNJ-31020028. The protocol was validated and the final product quality was analysed. *N*-[^11^C]Me-JNJ-31020028 was achieved with a radiochemical purity > 99%, RCY of 31% and molar activity of 156 GBq/μmol. The precursor *N*-Me-JNJ-31020028 was synthesised using batch synthesis and continuous flow methodology, with 43% and 92% yields, respectively. The studies executed in the continuous flow approach allowed the *N*-methylation of precursor **1** in a very short time (5 min of residence time) with complete conversion and high yields in which it was possible to obtain up to 1.4 mmol/h of productivity for *N*-Me-JNJ-31020028. These results are promising in implementing this methodology for PET tracer production. PET studies with *N*-[^11^C]Me-JNJ-31020028 were performed in healthy mice and confirmed the accumulation of the tracer in regions with known Y_2_ receptor density, such as the gut and four areas of the brain: the hypothalamus, striatum, amygdala and hippocampus. The tracer will be tested in pre-clinical models of brain disorders affecting memory, response to stress and emotional control, particularly on the autism spectrum.

## Figures and Tables

**Table 1:** Optimisation of the continuous flow synthesis of **2** in a tubular reactor (10 mL) ^(a)^.

Entry	Stoichiometry (Equiv. of CH_3_I)	Temperature (°C)	Residence Time (min)	Flow Rate (mL/min)	Conversion (%) ^(b),(c)^	Yield (%) ^(b),(c)^
1	1.5	25	25	0.4	82 ± 3.8	64 ± 7.2
2	1.5	25	10	1.0	99 ± 0.6	87 ± 3.5
3	1.5	25	5	2.0	99 ± 1.0	90 ± 1.5
4	1.5	25	3	3.3	99 ± 1.5	88 ± 2.5
5	1.5	40	5	2.0	97 ± 2.0	92 ± 1.5
6	3.0	25	5	2.0	98 ± 2.0	81 ± 3.2
7	3.0	40	5	2.0	98 ± 1.5	77 ± 7.6

^(a)^ NaH (0.038 M), CH_3_I (0.038 M or 0.075 M), A:B (1:1) volumetric ratio. ^(b)^ Values are expressed in mean ± SD, *n* = 3, *p* < 0.05 (Grubbs’ test was performed to verify outliers). ^(c)^ Obtained by HLPC analysis.

**Table 2:** Optimisation of the continuous flow synthesis of **2** in a microchip reactor (1.5 mL) ^(a)^.

Entry	Stoichiometry (Equiv. of CH_3_I)	Temperature (°C)	Residence Time (min)	Flow Rate (mL/min)	Conversion (%) ^(b),(c)^	Yield (%) ^(b),(c)^
1	1.5	25	5	0.3	66 ± 2.1	60 ± 4.9
2	1.5	40	5	0.3	94 ± 2.0	88 ± 0.6
3	3.0	40	5	0.3	95 ± 1.0	89 ± 1.5
4	CH_3_I (gas)	40	5	0.3	98 ± 3.2	67 ± 5.3

^(a)^ NaH (0.038 M), CH_3_I (0.038 M or 0.075 M), A: B (1:1) volumetric ratio. ^(b)^ Values are expressed in mean ± SD, *n* = 3, *p* < 0.05 (Grubbs’ test was performed to verify outliers). ^(c)^ Obtained by HLPC analysis.

**Table 3:** Optimisation of *N*-[^11^C]Me-JNJ-31020028 radiolabelling reaction conditions.

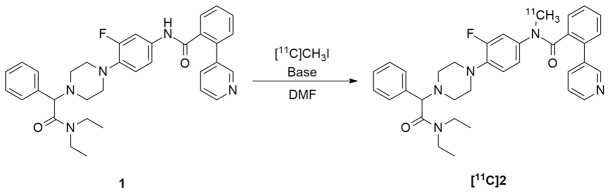						
Entry	Base	Temperature (°C)	Stirring	Reaction Time (min)	[^11^C]2 (%) ^(a),(b)^	RCY (d.c) (%) ^(b),(c)^
1 ^(d)^	NaH (0.02 mg/mL)	50	Yes	3	90.4 ± 0.65	41.8 ± 1.06
2	NaH (0.02 mg/mL)	50	Yes	3	85.4 ± 5.69	39.9 ± 0.68
3	NaH (0.02 mg/mL)	50	No	3	81.1 ± 7.77	37.4 ± 1.22
4	NaH (0.02 mg/mL)	50	Yes	5	78.4 ± 14.95	34.4 ± 5.06
5	NaOH (5M)	50	No	5	91.2 ± 3.04	38.3 ± 4.37
6	NaOH (5M)	50	Yes	5	68.7 ± 11.77	26.4 ± 3.66
7	NaOH (5M)	75	No	5	88.5 ± 6.30	41.0 ± 4.8
8	NaOH (5M)	75	Yes	5	80.3 ± 8.27	33.0 ± 7.83
9 ^(e)^	NaOH (5M)	rt	No	5	98.7 ± 1.14	31.3 ± 1.07 ^(f)^

^(a)^ Determined by HPLC analysis. ^(b)^ Values are expressed in mean ± SD, *n* = 3, *p* < 0.05 (Grubbs’ test was performed to verify outliers). Higher standard deviation values are due to variability in the equipment and process. ^(c)^ Radiochemical yield (decay corrected): the amount of activity in the product expressed as the percentage (%) of related starting activity ([^11^C]CO_2_) utilised in the process. ^(d)^ With DMF-THF mixture as solvent. ^(e)^ Reaction performed in a loop. ^(f)^ RCY (d.c.) after semipreparative HPLC purification.

**Table 4:** Quality control tests and specifications for *N*-[^11^C]-Me-JNJ31020028 injectable solution.

Tests	Specifications	*N*-[^11^C]-Me-JNJ31020028-1	*N*-[^11^C]-Me-JNJ31020028-2	*N*-[^11^C]-Me-JNJ31020028-3
**Appearance**	Clear, colourless solution	Comply	Comply	Comply
**pH after dilution**	4.5–8.5	6.4	6.4	6.5
**Chemical purity**				
*N*-Me-JNJ31020028	≥95%	100%	100%	100%
**Radiochemical purity**				
*N*-[^11^C]-Me-JNJ31020028	≥95%	99.2%	99.9%	99.4%
**Radionuclidic purity**				
Radionuclidic identification − Energy photons ϒ	The only gamma photons have energy of 0.511 MeV. A sum peak of 1.022 MeV may be observed	Comply	Comply	Comply
Half-life	19.9 to 20.9 min	20.1	20.2	20.2
**Residual Solvents**				
Ethanol	≤2500 mg/10 mL ^(a)^	491.2	900.8	504.2
Acetonitrile	≤4 mg/10 mL	2.9	2.0	0.8
**Biological Tests**				
Sterility ^(b)^	No evidence of microbial growth should be found	Comply	Comply	Comply
Bacterial endotoxins	≤175 IU/10 mL	Comply	Comply	Comply

^(a)^ According to Ph. Eur., maximum 2.5 g per administration taking the density (2.2.5) to be 0.790 g/mL [26]. ^(b)^ The product may be released for use before the completion of these tests.

## Data Availability

Data are contained within the article and in the Supplementary Materials.

## Supplementary Materials

The following supporting information can be downloaded at: https://www.mdpi.com/article/10.3390/ph17040474/s1, Figure S1: ^1^H NMR spectra of *N*-Me-JNJ-31020028 ang signal attribution; Figure S2: ^13^C NMR spectra of *N*-Me-JNJ-31020028 ang signal attribution; Figure S3: ^1^H-^1^H COSY spectra of *N*-Me-JNJ-31020028 and ^1^H signal attribution; Figure S4: NOESY spectrum of *N*-Me-JNJ-31020028. Figure S5: ^1^H-^13^C HSQC spectrum of *N*-Me-JNJ-31020028 and ^1^H and ^13^C signal attribution; Figure S6: Selected expansion of ^1^H-^13^C HMBC spectrum of *N*-Me-JNJ-31020028; Figure S7: HRMS spectrum of *N*-Me-JNJ-31020028; Figure S8: Analytical HPLC chromatogram of precursor JNJ-31020028 **1**; Figure S9: Representative analytical HPLC chromatogram of the reaction mixture obtained through continuous flow methodology; Table S1: In vivo biodistribution; Table S2: Whole body ex vivo biodistribution; Table S3: Brain ex vivo biodistribution.
