# Supplementary material for: Improved Chemical and Radiochemical Synthesis of Neuropeptide Y Y2 Receptor Antagonist N-Methyl-JNJ-31020028 and Preclinical Positron Emission Tomography Studies
Source: Pharmaceuticals (Basel). 2024 Apr 8;17(4):474. doi: 10.3390/ph17040474 (PMC11053772; doi:10.3390/ph17040474)
Supplement: Supplementary file 1 [file pharmaceuticals-17-00474-s001.zip › pharmaceuticals-2937642-supplementary.pdf]

Supporting information

Improved chemical and radiochemical synthesis of Neuropeptide Y Y_2 receptor antagonist *N*-Methyl-JNJ-31020028 and preclinical PET studies

Inês C. F. Fonseca, Mariana Lapo Pais, Fábio M. S. Rodrigues, José Sereno, Miguel Castelo-Branco, Cláudia Cavadas, Mariette M. Pereira and Antero J. Abrunhosa

Characterisation of *N*-Me-JNJ-31020028 ..... S2

- ^{1}H-NMR S2
- ^{13}C-NMR S3
- ^{1}H-^{1}H COSY (Correlated spectroscopy) S4
- NOESY spectrum. S4
- ^{1}H-^{13}C HSQC (Heteronuclear Single Quantum Coherence) S5
- ^{1}H-^{13}C HMBC (Heteronuclear Single Quantum Coherence) S5
- HRMS spectrum S6

HPLCs S6

Pre-clinical studies S7

Characterisation of *N*-Me-JNJ-31020028

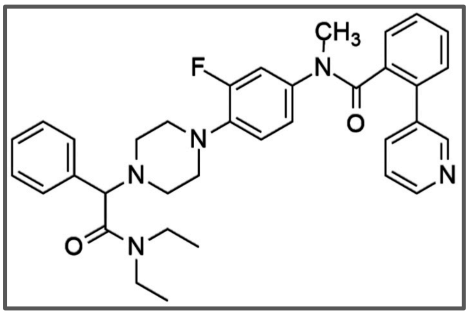

^{1}H-NMR (400 MHz, $CDCl_3$) δ/ppm: 8.51 (d, J = 4.0 Hz, 1H), 8.15 (d, J = 1.4 Hz, 1H), 7.5 (d, J = 7.2 Hz, 1H), 7.41 (d, J = 7.8 Hz, 1H), 7.31-7.23 (m, 5H), 7.21-7.18 (m, 1H), 7.02 (d, J = 7.2 Hz, 1H), 6.38 (t, J = 9.0 Hz, 1H), 5.84 (dd, J = 8.5, 2.4 Hz, 1H), 5.80 (dd, J = 13.3, 2.4 Hz, 1H), 4.15 (s, 1H), 3.39-3.30 (m, 2H), 3.13 (s, 3H), 3.23-3.08 (m, 2H), 2.92 (brs, 4H) 2.61-2.52 (m, 4H), 1.00 (t, J = 7.1 Hz, 3H), 0.96 (t, J = 7.1 Hz, 3H). ^{13}C-NMR (100 MHz, $CDCl_3$) δ/ppm: 170.6, 169.8, 154.3 (d, J = 248.1 Hz), 149.5, 148.2, 138.5 (d, J = 8.5 Hz), 136.7 (d, J = 9.5 Hz), 136.2, 135.8, 135.8, 135.0, 129.9, 129.5, 129.3, 129.2, 128.7, 128.7, 128.3, 123.0, 122.0 (d, J = 2.9 Hz), 117.9 (d, J = 3.4 Hz), 113.9 (d, J = 28.2 Hz), 70.9, 51.2, 50.5, 41.6, 40.8, 37.1, 12.9, 12.4. HRMS (ESI): *m/z* calcd. for $C_{35}H_{39}FN_5O_2^+$ $[M+H]^+$: 580.3082, found: 580.3082.

- ^{1}H-NMR

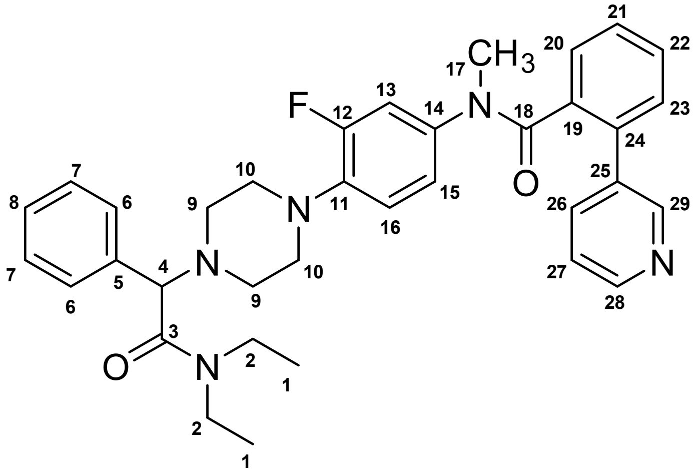

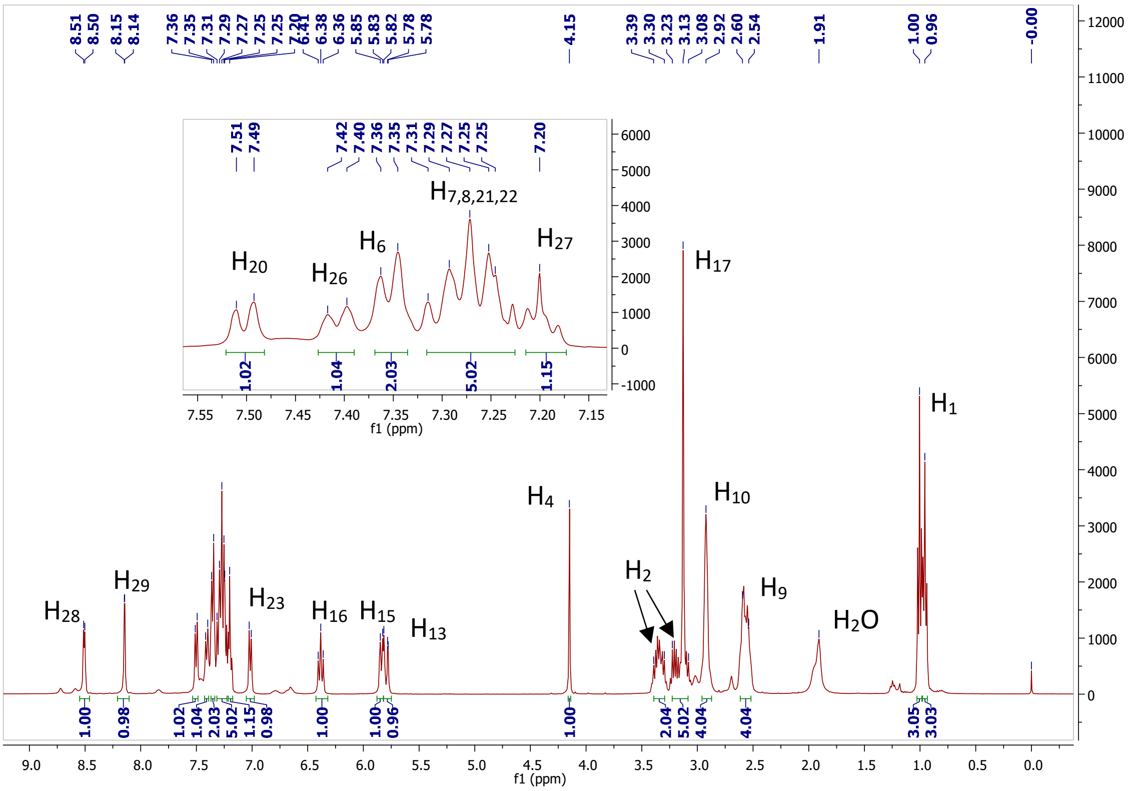

Figure S1- ^{1}H NMR spectra of N-Me-JNJ-31020028 ang signal attribution.

- ^{13}C-NMR

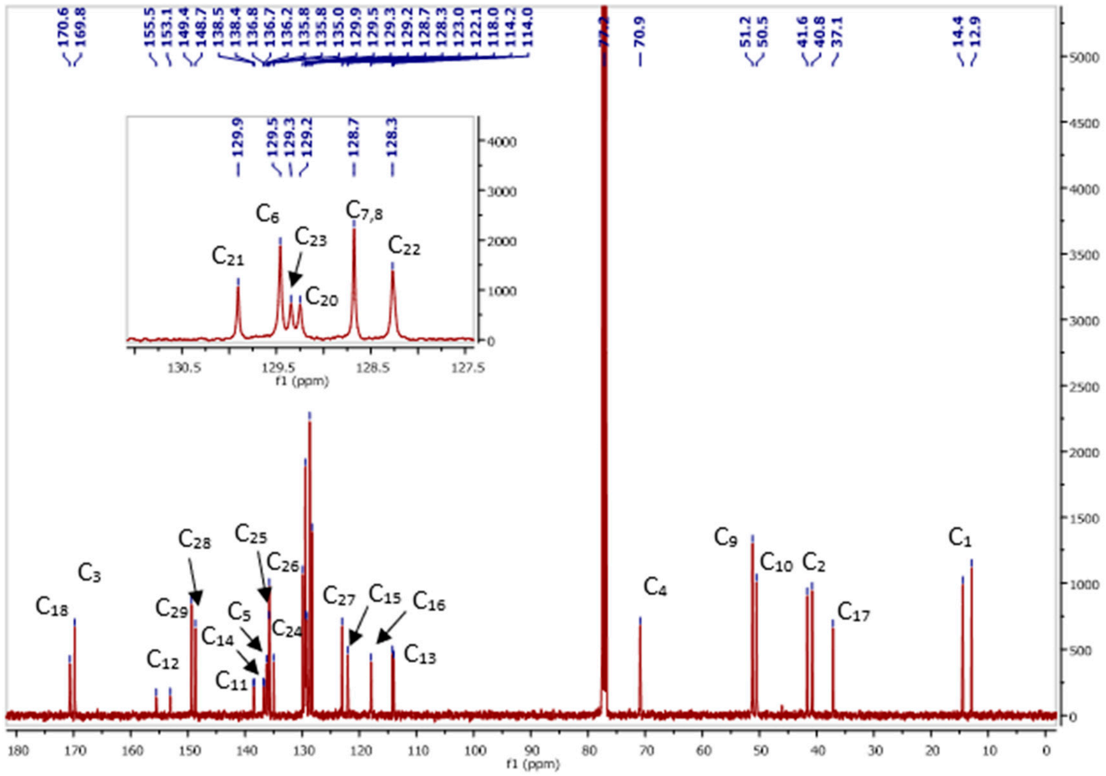

Figure S2- ^{13}C NMR spectra of N-Me-JNJ-31020028 ang signal attribution.

- ^{1}H-^{1}H COSY (Correlated spectroscopy)

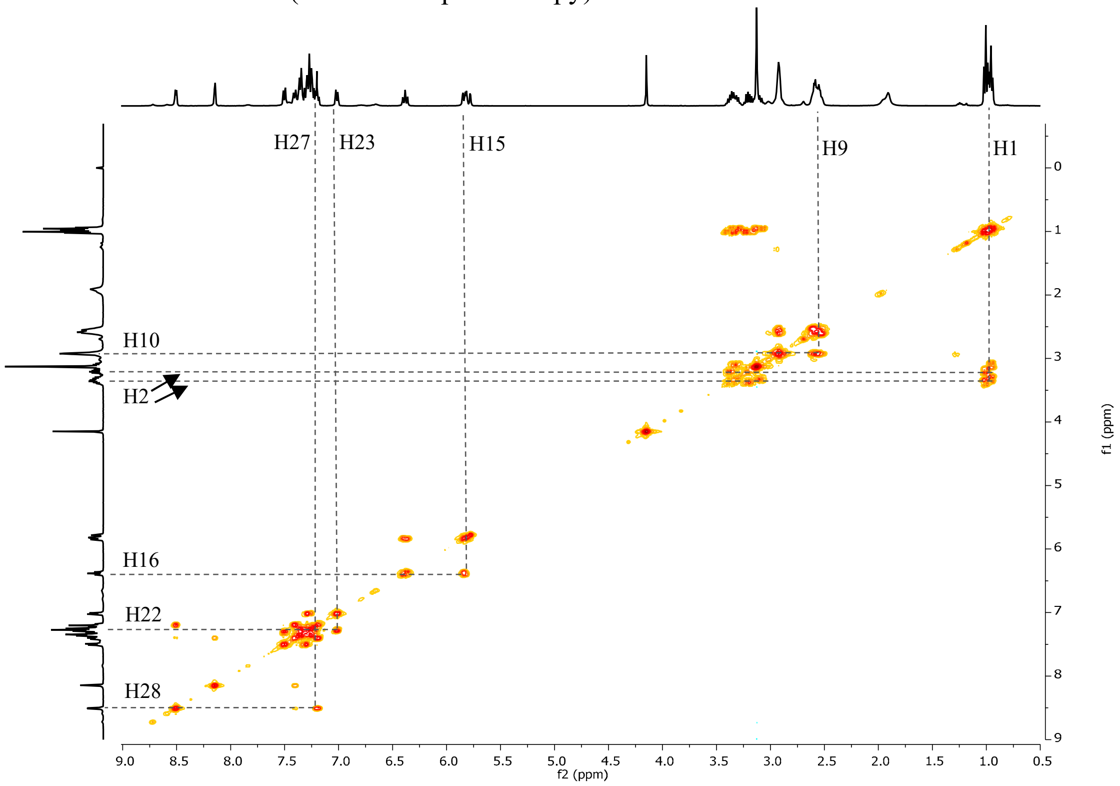

Figure S3- ^{1}H-^{1}H COSY spectra of N-Me-JNJ-31020028 and ^{1}H signal attribution.

- NOESY Spectrum

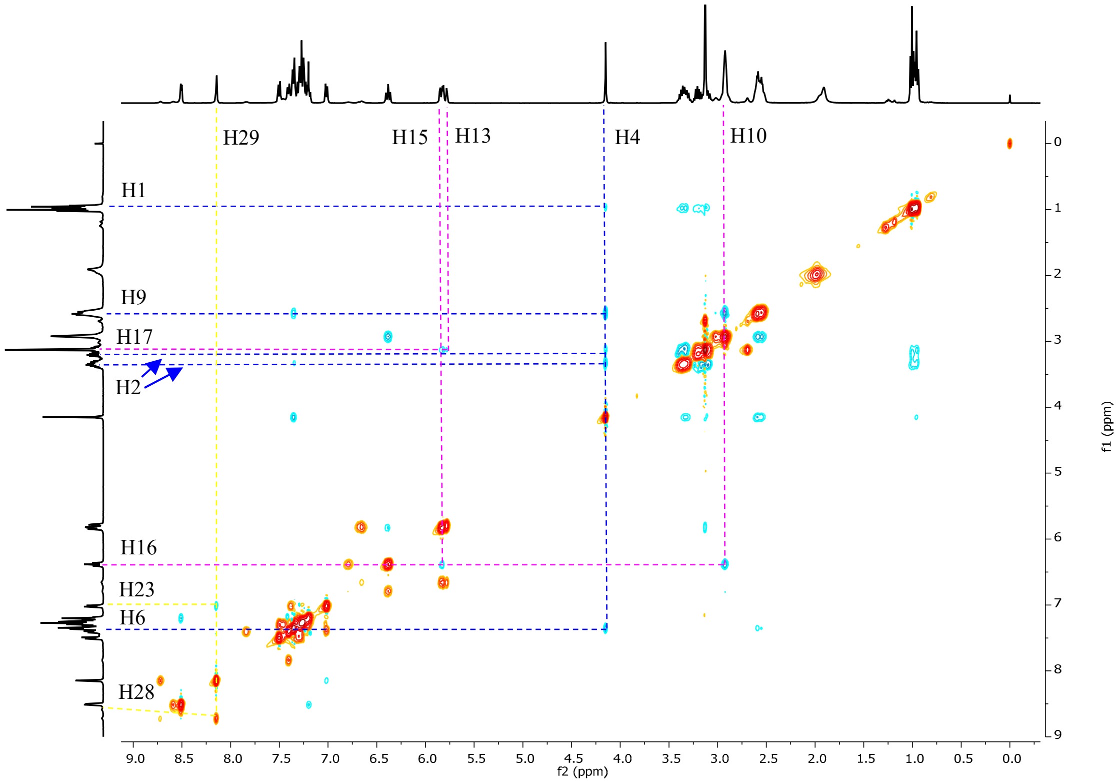

Figure S4- NOESY spectrum of *N*-Me-JNJ-31020028. The most relevant correlations regarding the protons H4, H13, H15, H16 and H29 are presented.

- ^{1}H-^{13}C HSQC (Heteronuclear Single Quantum Coherence)

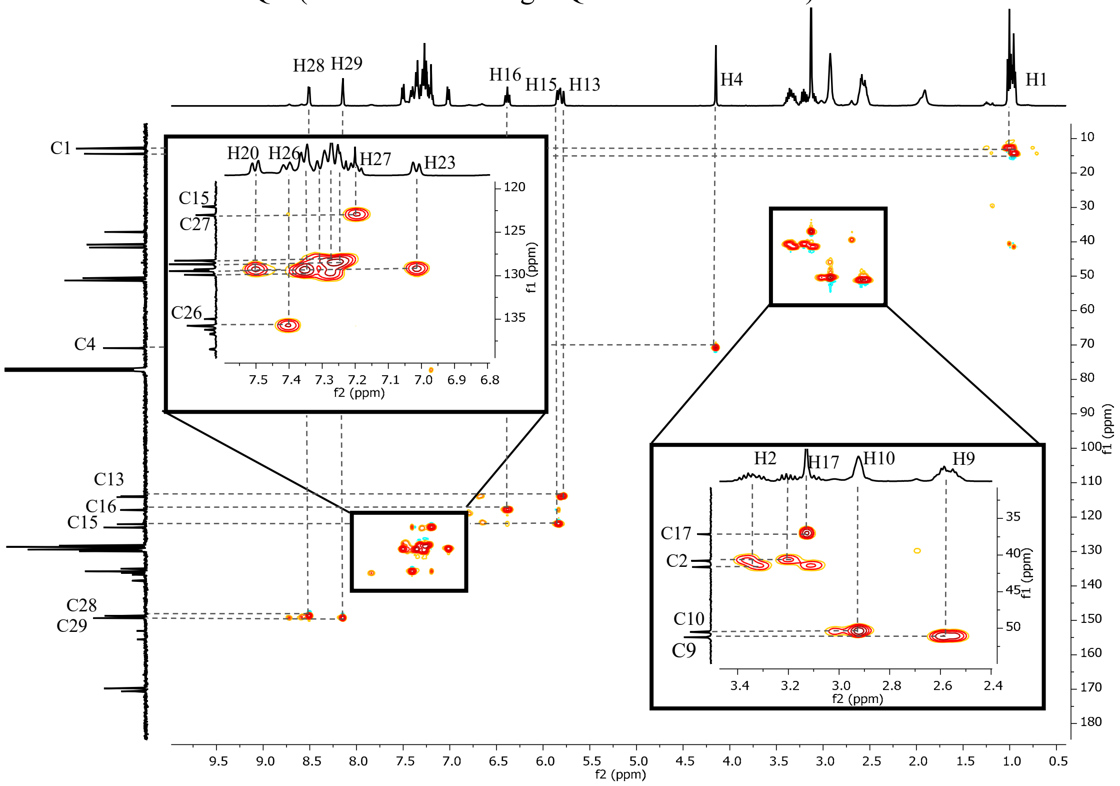

Figure S5- ^{1}H-^{13}C HSQC spectrum of *N*-Me-JNJ-31020028 and ^{1}H and ^{13}C signal attribution. Inset: selected expansions of the ^{1}H-^{13}C HSQC spectrum between δ 2.4 to 3.4 ppm and δ 6.8 to 7.6 ppm.

- ^{1}H-^{13}C HMBC (Heteronuclear Single Quantum Coherence)

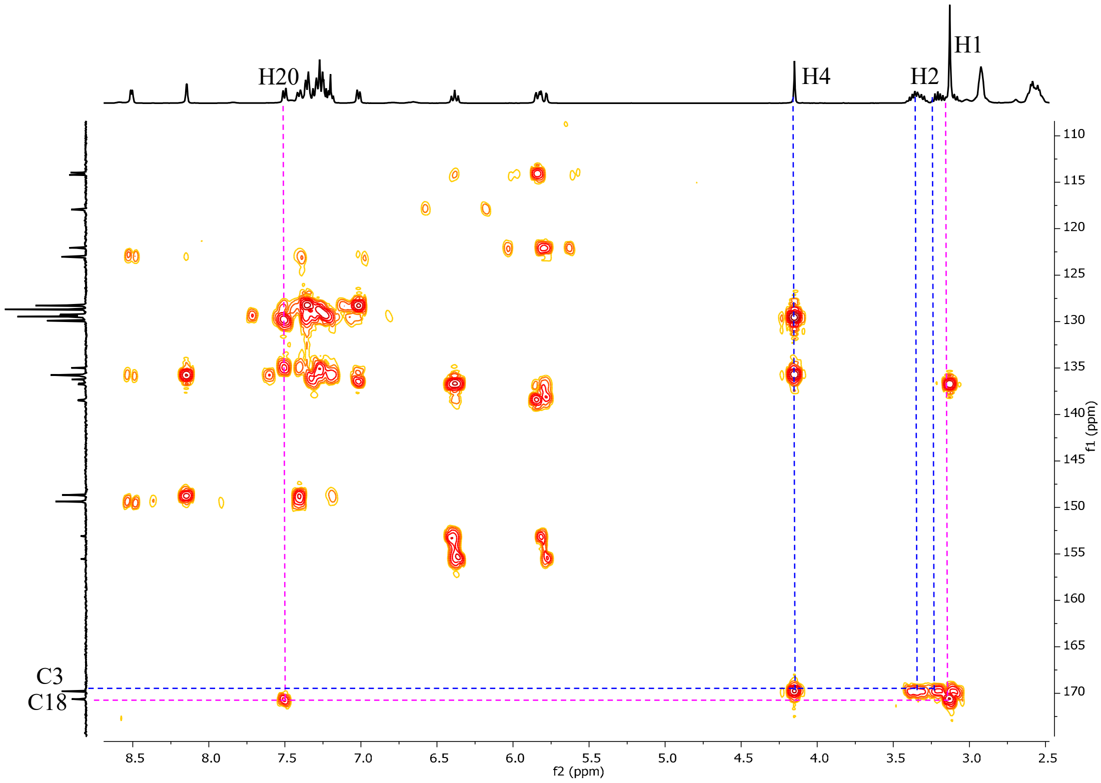

Figure S6- Selected expansion of ^{1}H-^{13}C HMBC spectrum of *N*-Me-JNJ-31020028. The most relevant correlations regarding the carbons C3 and C18 are presented.

- HRMS

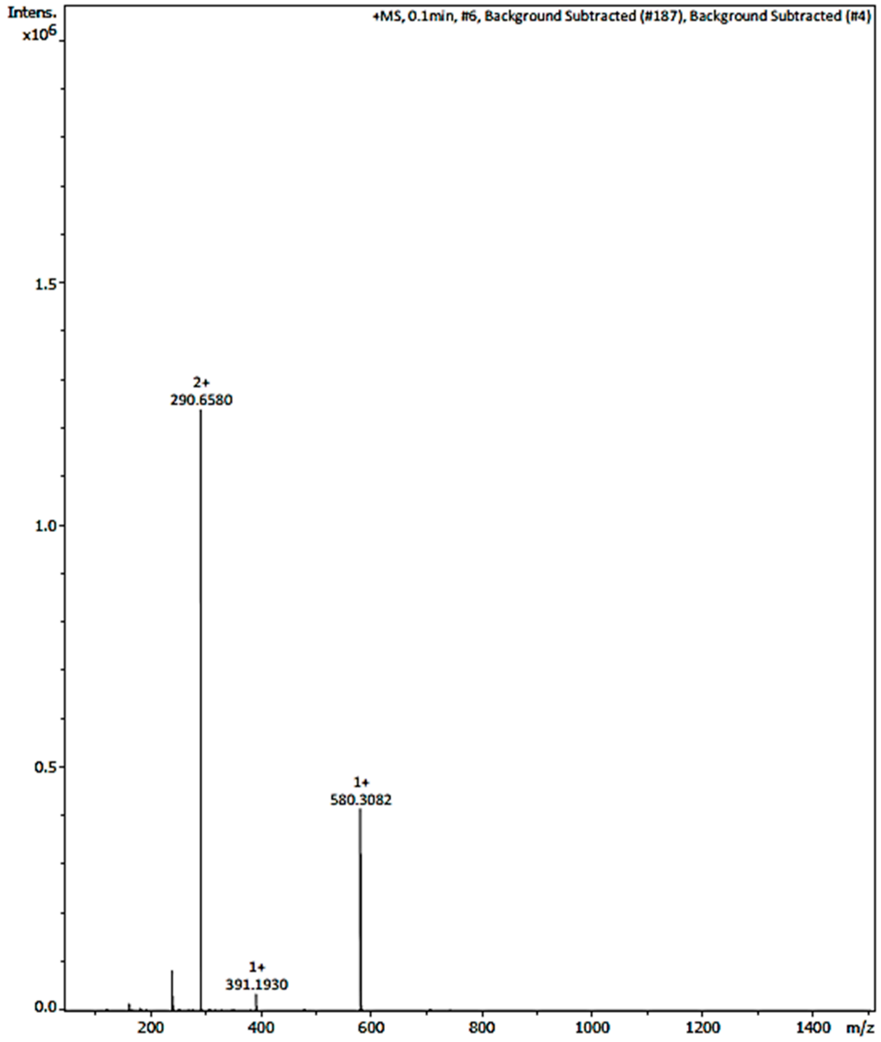

Figure S7- HRMS spectrum of *N*-Me-JNJ-31020028.

HPLCs

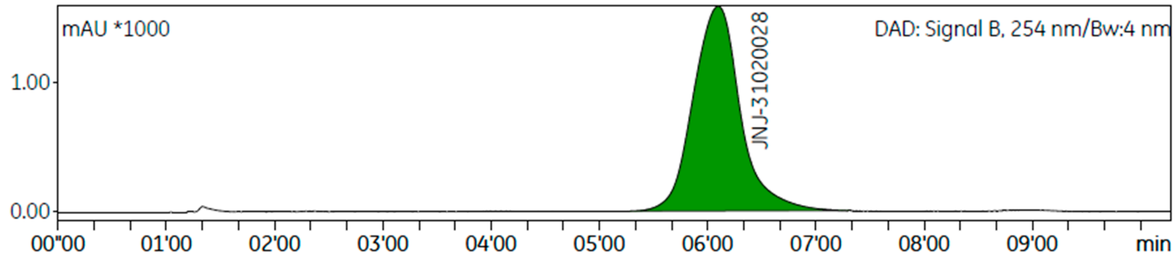

Figure S8- Analytical HPLC chromatogram of precursor JNJ-31020028 **1**. Rt: 6.06 min.

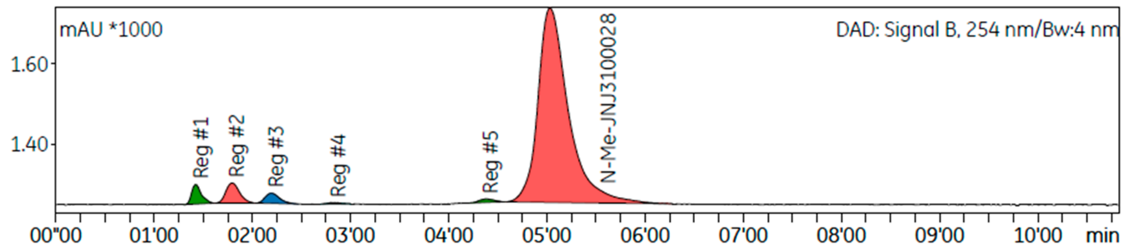

Figure S9- Representative analytical HPLC chromatogram of the reaction mixture obtained through continuous flow methodology. Retention times: Reg#1: 1.26 min; Reg#2: 1.48 min; Reg#3: 2.12 min; Reg#4: 2.50 min; Reg#5: 4.23 min; *N*-Me-JNJ-31020028: 5.02 min.

Pre-clinical studies

Table S1- *In vivo* biodistribution. Values are expressed as percentage injected dose *per* gram (%ID/g) of body weight (n = 7).

Region	Mean	SEM
Brain	0.5187	0.07698
Gut	12.57	1.217

Table S2- Whole body *ex vivo* biodistribution. Values are expressed as percentage injected dose *pe*r gram (%ID/g) of tissue (n = 5).

Region	Mean	SEM
Blood	3.661	1.545
Urine	84.43	59.41
Liver	14.33	6.39
Spleen	1.504	0.5483
Lung	0.5568	0.04036
Heart	1.394	0.701
Kidney	1.986	0.4916
Stomach	3.082	0.7135
Small intestine	98.64	25.57
Large intestine	2.18	0.2095
Muscle	0.9318	0.193

Faeces	3.149	1.092
Bile	250.1	23.97
Brain	0.6362	0.06454

Table S3- Brain *ex vivo* biodistribution. Values are expressed as percentage injected dose *per* gram (%ID/g) of tissue (n = 5).

Region	Mean	SEM
Cerebellum	0.646	0.09664
Hypothalamus	1.03	0.1126
Amygdala	0.9318	0.08237
Striatum	1.013	0.1068
Pre-frontal cortex (PFC)	0.636	0.05916
Hippocampus	0.8976	0.09311
